# The persistent viral infections in the development and severity of myalgic encephalomyelitis/chronic fatigue syndrome

**DOI:** 10.1186/s12967-023-03887-0

**Published:** 2023-01-18

**Authors:** Santa Rasa-Dzelzkaleja, Angelika Krumina, Svetlana Capenko, Zaiga Nora-Krukle, Sabine Gravelsina, Anda Vilmane, Lauma Ievina, Yehuda Shoenfeld, Modra Murovska, the VirA project

**Affiliations:** 1 Institute of Microbiology and Virology, Rīga Stradiņš University, Riga, Latvia; 2 Faculty of Medicine, Department of Infectology, Rīga Stradiņš University, Riga, Latvia; 3 Zabludowicz Center for Autoimmune Diseases, Sheba Medical Centre, Ramat Gan, Israel

**Keywords:** Myalgic encephalomyelitis/chronic fatigue syndrome,, HHV-6A, HHV-6B, HHV-7, Human parvovirus B19

## Abstract

**Background:**

Myalgic encephalomyelitis/chronic fatigue syndrome (ME/CFS) is a multifactorial disease with an unexplained aetiology in which viral infections are possible trigger factors.

The aim of this study was to determine the involvement of human herpesvirus (HHV)-6A/B, HHV-7, and parvovirus B19 (B19V) in the etiopathogenesis of ME/CFS.

**Methods:**

200 patients with clinically diagnosed ME/CFS and 150 apparently healthy individuals were enrolled in this study. Single-round, nested, and quantitative real-time polymerase chain reactions (PCR) were used to detect the presence and load of HHV-6A/B, HHV-7, and B19V. HHV-6A and HHV-6B were distinguished by PCR and restriction analysis. Immunoenzymatic assays were applied to estimate the presence of virus-specific antibodies and the level of cytokines.

**Results:**

HHV-6A/B, HHV-7, and B19V specific antibodies were detected among patients and healthy individuals in 92.1% and 76.7%, 84.6% and 93.8%, and 78% and 67.4% of cases. HHV-6B had 99% of HHV-6 positive patients.

Latent HHV-6A/B, HHV-7, and B19V infection/co-infection was observed in 51.5% of the patients and 76.7% of the healthy individuals, whereas active–45% of the ME/CFS patients and 8.7% of healthy individuals. HHV-6A/B load in patients with a persistent infection/co-infection in a latent and active phase was 262 and 653.2 copies/10^6^ cells, whereas HHV-7 load was 166.5 and 248.5 copies/10^6^ cells, and B19V-96.8 and 250.8 copies/10^6^ cells, respectively.

ME/CFS patients with persistent infection in an active phase had a higher level of pro-inflammatory cytokines (interleukin(IL)-6, tumor necrosis factor-alpha(TNF-α) and IL-12) and anti-inflammatory (IL-10) than with a persistent infection in a latent phase. A significant difference was revealed in the levels of TNF-α, IL-12, and IL-10 among the patient groups without infection, with latent infection/co-infection, active single, double and triple co-infection. The levels of TNF-α, IL-12, and IL-10 are significantly higher in patients with severe compared with a moderate course of ME/CFS.

**Conclusions:**

Significantly more persistent HHV-6A/B, HHV-7, and B19V infection/co-infection in an active phase with a higher viral load and elevated levels of pro- and anti-inflammatory cytokines among patients with ME/CFS than healthy individuals indicate the importance of these infections/co-infections in ME/CFS development. The presence of these infections/co-infections influences the ME/CFS clinical course severity.

## Background

ME/CFS is a chronic, complex disease involving central nervous system and immune system disorders, cell energy metabolism and ion transport dysfunction, as well as cardiovascular abnormalities [1]. The illness mainly is characterized by severe chronic fatigue, including such clinical symptoms as tender cervical or axillary lymph nodes, muscle pain, joint pain without swelling or redness, post-exertional malaise for more than 24 h, impaired memory/concentration, headaches, sore throat and un-refreshing sleep [1, 2].

The reported prevalence of ME/CFS depends on the applied criteria for diagnosis and it is determined from 0.76% of clinically diagnosed up to 3.48% of the self-reported population [3]. Reporting ME/CFS prevalence of 0.89%, researchers urge to seek an objective diagnostic tool for ME/CFS [4]. Still, there is no consensus on a single case definition for this disease. Diagnosis is based on differential diagnostics and clinical symptoms, therefore it is necessary to identify specific biomarkers for ME/CFS. However, currently, there are no effective and standardized diagnostic tests, prophylactic and treatment strategies for this disease [5, 6].

Viral infections have been considered as one of the potential etiological factors for ME/CFS, which accompanied by immune disturbances can facilitate the maintenance of disease symptoms [6–9]. Many patients confirm an onset of ME/CFS with flu-like symptoms. Moreover, the observed immune abnormalities could be caused by a viral infection or by the viral infection causedimmune disturbances. Still, the role of viral infections in ME/CFS remains obscure [10–12].

While some researchers find no association of HHV-6A/B, HHV-7, and B19V infection with ME/CFS etiopathogenesis [13, 14], others report that the reactivation of these viruses could serve as an objective biomarker [15–18], supported by recent evidence of Epstein-Barr virus (EBV) and HHV-6A role in ME/CFS [8]. Also, immune system disorders are determined in various studies by the analysis of changes in several cytokine productions in patients with ME/CFS [19, 20]. Therefore, it is important to conduct studies in order to clarify the role of these viruses in ME/CFS, as well as to determine etiological, progression, maintenance mechanisms, and biomarkers for this disease.

The aim of the study was to determine the involvement of HHV-6A/B, HHV-7, and B19V in the etiopathogenesis of ME/CFS.

## Methods

Two hundred patients [130 (65%) female and 70 (35%) male, mean age 38 ± 12] with a clinically diagnosed ME/CFS corresponding to 1994 Fukuda Centers for Disease Control and Prevention criteria and 150 age and gender matched apparently healthy individuals were included in this cross-sectional study, aiming to determine the involvement of HHV-6A/B, HHV-7, and B19V in the etiopathogenesis of ME/CFS.

All the patients were evaluated by interviews with questionnaires. The symptom pattern in ME/CFS patients was examined using adapted semi-structured interview questions [21]. Sleep disturbances were evaluated with a self-reported questionnaire—Athens Insomnia Scale 8 [22]. Based on the questionnaire, points were given and answers were graded defining the disease course as mild (0–7 points), moderate (8–12 points), or severe (13–15 points).

DNA was isolated from peripheral blood by phenol–chloroform extraction method and from blood plasma samples – using QIAamp DNA Blood Kit, (Qiagen GmbH, Germany), according to manufacturer’s instruction. DNA concentration was measured spectrophotometrically with “NanoDrop” spectrophotometer and quality was assured by β-globin PCR based on Vandamme et al., 1995 [23].

Nested PCR (nPCR) was used to amplify HHV-6A/B, HHV-7, and B19V-specific genomic sequences in the DNA isolated from the peripheral blood (a marker of persistent infection) and cell-free blood plasma (a marker of an active infection). The detection of HHV-6A/B (U3 gene) and HHV-7 (U10 gene) genomic sequences was performed in accordance with earlier published approaches [24, 25], respectively. HHV-6A/B and HHV-7 genomic DNAs (Advanced Biotechnologies Inc, Columbia, MD, USA) were used as positive controls. The sensitivity of HHV-6A/B-specific primers was three copies and HHV-7–one copy per reaction [26, 27] Amplification conditions for HHV-6A/B PCR both cycles were following: initial denaturation–3 min, 95 °C; amplification (30 cycles:1 min, 94 °C; 1 min, 57 °C; 1 min, 72 °C); final synthesis–7 min, 72 °C. Whereas for first and second PCR cycles of HHV-7 detection—initial denaturation–4 min, 94 °C; amplification (30 cycles: 1 min, 94 °C; 2 min, 60 °C for cycle 1 and 2 min, 55 °C for cycle 2; 2 min, 72 °C); final synthesis – 7 min, 72 °C.

The presence of the B19V genomic sequence was determined according to Barah et al. [28], using primers complementary to the NS1 gene. Previously confirmed viremic serum DNA was used as a positive control. The sensitivity of primers was 1–10 copies per reaction [28]. B19V amplification conditions for both cycles were: initial denaturation–6 min, 95 °C; amplification (40 cycles: 30 s, 95 °C; 30 s, 55 °C; 30 s, 72 °C); final synthesis–7 min, 72 °C.

HHV-6A and HHV-6B were differentiated according to Lyall and Cubie [29]. Amplification conditions for both cycles were: initial denaturation–5 min, 95 °C; amplification (30 cycles: 1 min, 94 °C; 1 min, 60 °C; 1 min, 72 °C); final synthesis –10 min, 72 °C. Following nPCR, amplification products were digested with HindIII restriction endonuclease (Thermo Scientific, USA) which cleaves HHV-6B 163 bp amplification product into 66 bp and 97 bp fragments, whereas does not cleave HHV-6A. Electrophoretic analysis was done in 1.7% agarose gel to separate and identify the DNA fragments amplified by PCR and those mentioned above of following size: HHV-6A/B–258 bp; HHV-7–124 bp and B19V–103 bp. Results were visualised using UVP BioSpectrum MultiSpectral Imaging System (United Kingdom).

Viral load was estimated using DNA extracted from peripheral blood by real-time PCR according to the manufacturer’s instructions. HHV-6A/B load was determined with HHV-6 Real-TM Quant and B19V–with Parvovirus B19 Real-TM Quant kit (Sacace Biotechnologies, Italy). HHV-7 load was detected using Human Herpes Virus 7 genomes genesig kit (Primerdesign, United Kingdom) and in-house real-time PCR amplifying HHV-7 U90 and PI15 gene sequences based on a previous report by Prusty et al. [30]. The amplified data were analysed using BioRad CFX Manager Software Version 3.1.1517.0823.The presence of virus-specific Immunoglobulin (Ig)M and IgG class antibodies in blood plasma was detected using commercially available kits according to manufacturer’s protocol. IgM and IgG class antibodies against HHV-6A/B were detected with HHV-6 IgM and HHV-6 IgG enzyme linked immunosorbent assay (ELISA) kits (Panbio, Australia) and HHV-6 IgG Antibody ELISA kit (Advanced Biotechnologies, Columbia MD, USA). HHV-7 specific IgG class antibodies were analysed with the immunofluorescence method using the HHV-7 IgG IFA Kit (Advanced Biotechnologies, Columbia MD, USA). B19V-specific IgM and IgG class antibodies were estimated with Biotrin Parvovirus B19 Enzyme Immunoassay (Biotrin Ltd, Dublin, Ireland).

The determination of cytokine level in blood plasma was performed according to the manufacturer’s protocol. IL-6 level was detected with eBioscience Human IL-6 Platinium ELISA (eBioscience Europe/International, Austria), IL-10 level – using eBioscience Human IL-10 Platinium ELISA (eBioscience Europe/International, Austria), IL-12 (p70) level–with eBioscience Human IL-12p70 Platinium ELISA (eBioscience Europe/International, Austria), and TNF-α level–using Biorbyt Human TNFα ELISA kit (Biorbyt, United Kingdom). Absorbance was measured with microplate reader at 450 nm and concentration calculated.

The statistical analysis was done by GraphPad Prism 7.0 program. Discrete variables were described as numbers and percentage, and difference in frequency of gender, virus-specific antibodies, and virus presence markers between groups was estimated using Chi-square and Fisher´s exact tests as appropriate. Continuous variables were expressed as average ± standard deviation (SD) or median (interquartile range–IQR). Considering data distribution, viral loads and cytokine levels were analysed with Analysis of variance–ANOVA and Mann–Whitney nonparametric tests. A value of p ≤ 0.05 was considered to be statistically significant.

## Results

### ME/CFS patients

**Fig. 1:**
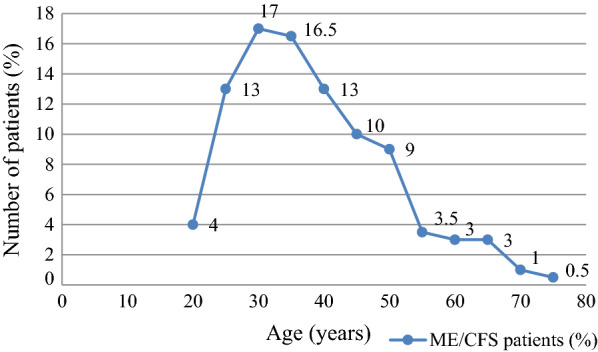
Age distribution among patients with myalgic encephalomyelitis/chronic fatigue syndrome

Out of 200 patients with ME/CFS enrolled in this study 65% (130/200) were female and 35% (70/200) were male (p < 0.0001). The mean (± SD) age for all patients was 38 ± 12 years. The age distribution is shown as a frequency of patients divided into 12 groups 20–75 years (with 5 year difference) (Fig. 1). 79% of patients were between age of 25–50 years.

**Fig. 2:**
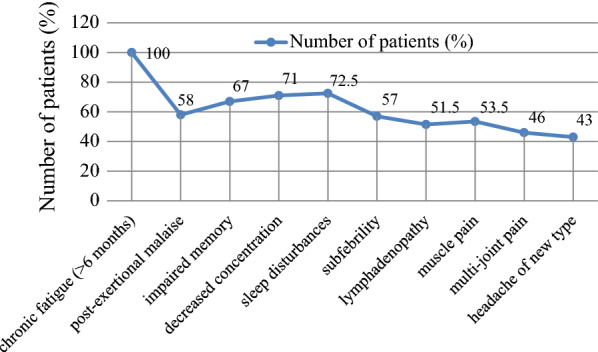
Frequency of typical symptoms of myalgic encephalomyelitis/chronic fatigue syndrome

The frequency of ME/CFS typical symptoms in 200 patients is depicted in Fig. 2. According to diagnostic criteria all patients experienced unexplained chronic fatigue lasting for more than 6 months (p < 0.0001) and at least four of the symptoms described below. Impaired memory, decreased concentration, and sleep disturbances were the most frequently observed symptoms in patients with ME/CFS. Impaired memory was present significantly more than muscle pain (p = 0.0435), lymphadenopathy (p = 0.0113), multi-joint pain (p = 0.0001), and headache of a new type (p < 0.0001). Decreased concentration was present in more patients than subfebrility (p = 0.0315), lymphadenopathy (p = 0.0005), muscle pain (p = 0.0025), multi-joint pain (p < 0.0001) and headache of a new type (p < 0.0001). Sleep disturbances were observed more frequently than post-exertional malaise (p = 0.0226), subfebrility (p = 0.0113), lymphadenopathy (p = 0.0001), muscle pain (p = 0.0007), multi-joint pain (p < 0.0001) and a new type of headache (p < 0.0001).

**Fig. 3:**
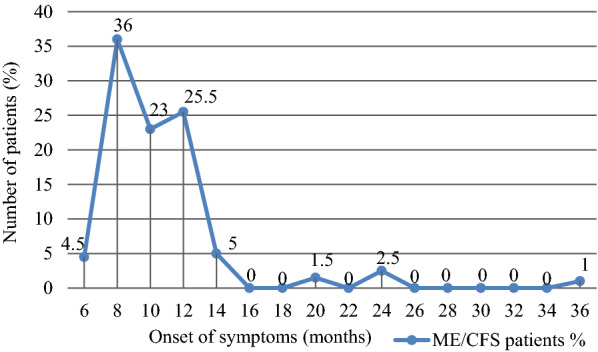
Onset time (in months before inclusion in the study) of myalgic encephalomyelitis/chronic fatigue syndrome typical symptoms

All included patients reported that the onset of ME/CFS symptoms occurred 6–36 months before inclusion in this study, with a mean (± SD) 10.2 ± 4.2 months. In 85% of patients, the onset of ME/CFS symptoms occurred 8–12 months before the inclusion in the study (Fig. 3).

### Presence of virus-specific antibodies

HHV-6A/B specific antibodies were detected in 92.1% (151/164) of analysed ME/CFS patients and 76.7% (69/90) apparently healthy individuals’ blood plasma samples (p = 0.0009). Anti-HHV-6A/B IgG class antibodies were found in 90.9% (149/164) patients and 76.7% (69/90) apparently healthy individuals (p = 0.0026), though IgM class antibodies were found in 6.1% (10/164) patients and 2.2% (2/90) apparently healthy individuals (p = 0.2227).

HHV-7 specific IgG class antibodies were detected in 84.6% (11/13) of the patients with ME/CFS and in 93.8% (30/32) of the apparently healthy individuals (p = 0.5672).

B19V-specific IgG class antibodies were detected in the blood plasma of 70% (140/200) of the patients with ME/CFS and in 67.4% (60/89) of the healthy individuals (p = 0.6803). None of the healthy individuals had B19V-specific IgM class antibodies, though 8% (16/200) of ME/CFS patients had IgM class antibodies (p = 0.0038).

### Presence of virus genomic sequences

**Fig. 4:**
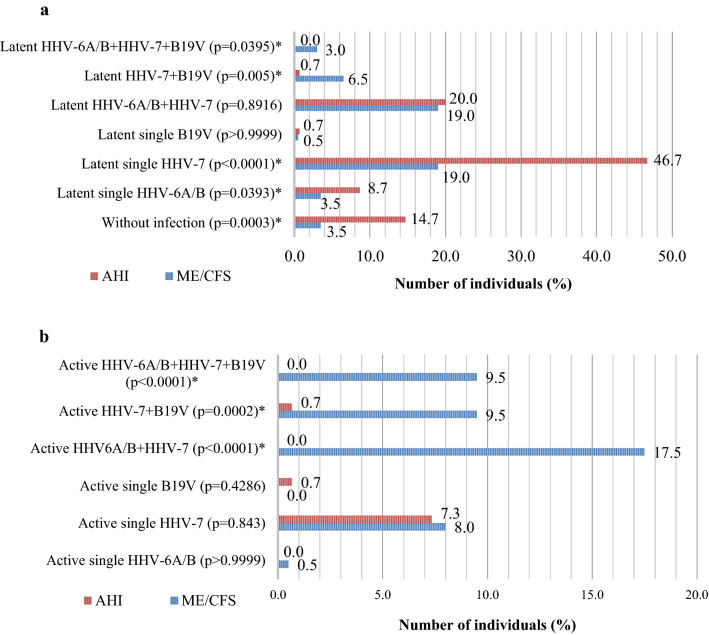
Frequency of persistent HHV-6A/B, HHV-7 and B19V infection/co-infection (%) in latent (**a**) or active (**b**) phase. HHV-human herpesvirus, B19V–human parvovirus B19, AHI–apparently healthy individuals. * statistically significant (p < 0.05)

Using nPCR markers of persistent viral infection/co-infection were revealed in 96.5% (193/200) of the patients with ME/CFS and in 85.3% (128/150) of apparently healthy individuals (p = 0.0003). From them, markers of latent infection/co-infection (virus genomic sequences in DNA from peripheral blood leukocytes) were observed in 51.5% (103/200) of the patients and in 76.7% (115/150) of the healthy individuals (p < 0.0001). Whereas markers of active infection/co-infection (virus genomic sequences in DNA from blood plasma) were detected in 45% (90/200) of the patients with ME/CFS and 8.7% (13/150) of the healthy individuals (p < 0.0001). HHV-6A/B, HHV-7, and B19V genomic sequences were not detected in 3.5% (7/200) of the patients and 14.7% (22/150) of the healthy individuals (p = 0.0003). Figures 4a, b shows the frequency (%) of markers for HHV-6A/B, HHV-7, and B19V infection/co-infection in latent or active phase in the groups of the patients with ME/CFS compared with the healthy individuals. HHV-6A was detected in one and HHV-6B in the rest 99% (105 HHV-6 positive) of the analysed patients with ME/CFS (p < 0.0001).

### Viral load in patients with co-infection

**Fig. 5:**
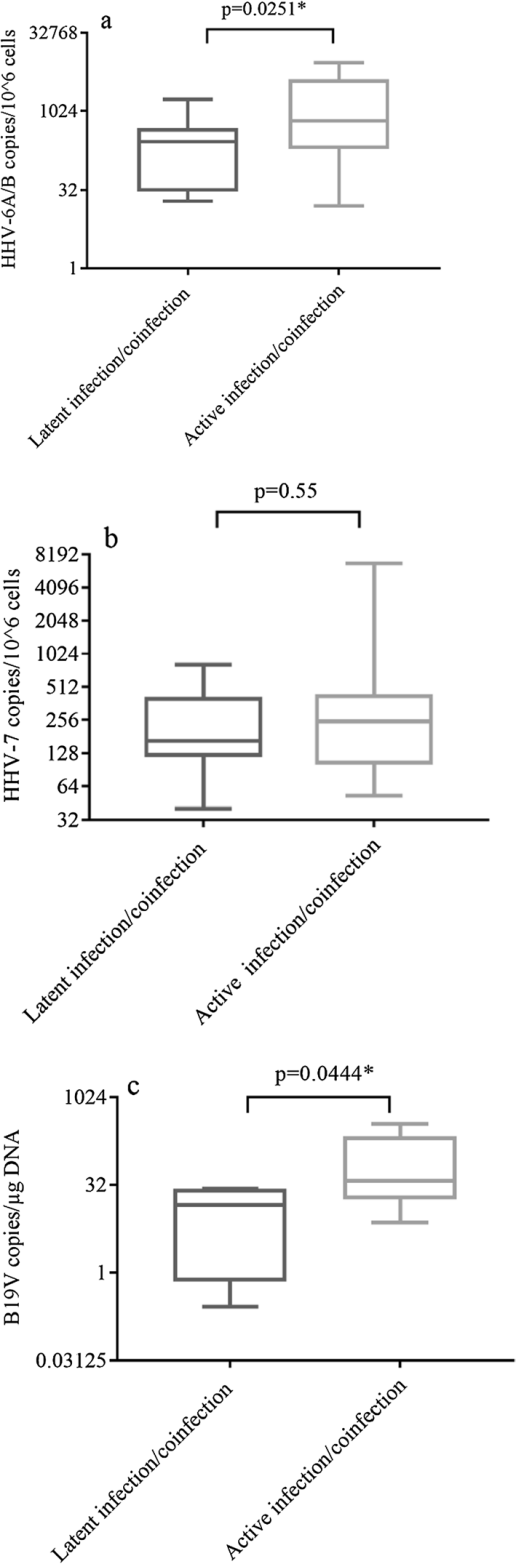
HHV-6A/B (**a**), HHV-7 (**b**) and B19V (**c**) viral load in ME/CFS patients with persistent infection/co-infection in latent and active phase. * statistically significant (p < 0.05)

Elevated HHV-6A/B load (> 10 copies/10^6^ cells) was detected in 50% of ME/CFS patients with a persistent infection/co-infection in the latent and in 79.6% of the patients in active phase (p = 0.0028). The median HHV-6A/B load in patients with a persistent infection/co-infection in the latent phase was (IQR) 262 (474–29.7) copies/10^6^ cells, whereas in the active phase–653.2 (4136–190.5) copies/10^6^ cells (p = 0.0251) (Fig. 5a).

Similarly, 58.3% of the patients with a persistent infection/co-infection in latent phase and 76.2% in active phase had elevated HHV-7 load (> 10 copies/10^6^ cells) (p = 0.0209). HHV-7 load was 166.5 (398.6–123.8) copies/10^6^ cells in patients with a persistent infection/co-infection in the latent phase and 248.5 (422–105.6) copies/10^6^ cells in ME/CFS patients with active infection (p = 0.55) (Fig. 5b).

Elevated B19V load was detected in 21.9% of the patients with a persistent.

infection/co-infection in latent and 32.5% of patients in active phase (p = 0.4286).

In patients with a persistent infection/co-infection in latent phase B19V load was 14.7 (27.4–0.7) copies/µg DNA (96.8 copies/10^6^ cells) and in the patients with infection in active phase–38 (217.8–18) copies/µg DNA (250.8 copies/10^6^ cells) (p = 0.0444) (Fig. 5c).

### Cytokine level in ME/CFS patients with viral infection/co-infection

**Fig. 6:**
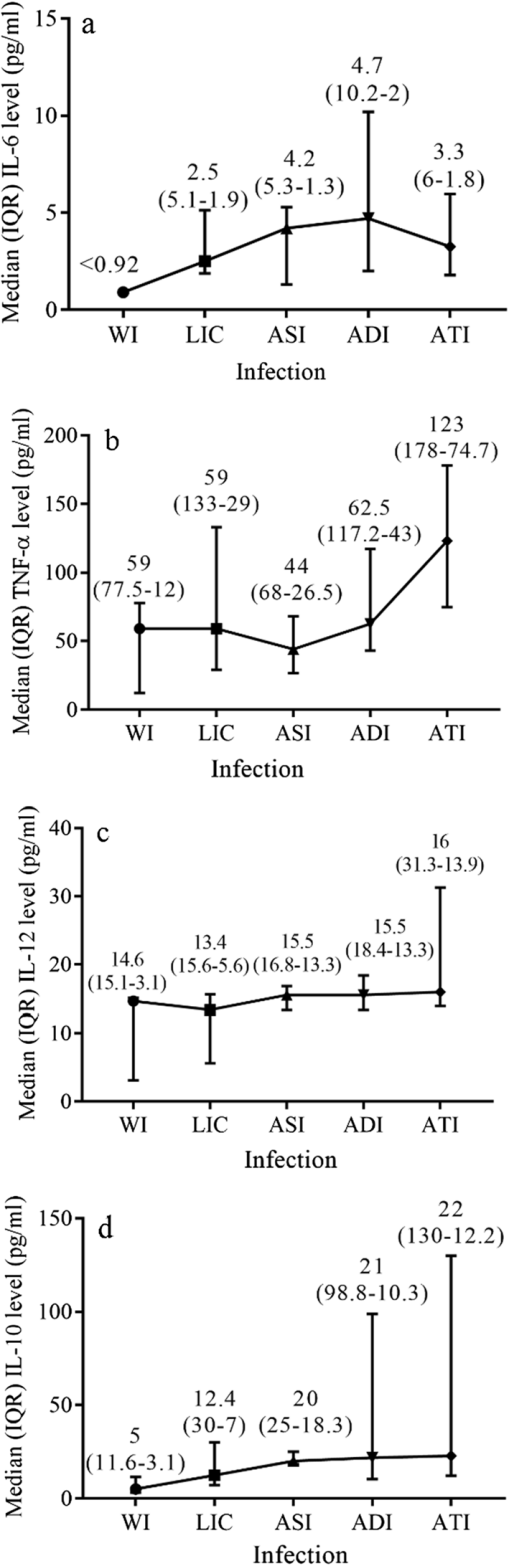
Median (IQR) IL-6 (**a**), TNF-α (**b**), IL-12 (**c**) and IL-10 (**d**) levels in ME/CFS patients with and without persistent infection/co-infection. WI-without infection, LIC–latent infection/co-infection, ASI–active single infection, *ADI* active double infection, *ATI* active triple infection

The data distribution was skewed, therefore median (IQR) values were suitable for displaying results of cytokine level. Median (IQR) levels of IL-6, TNF-α, IL-12, and IL10 in ME/CFS patients with persistent HHV-6A/B, HHV-7, B19V infection, and/or co-infection in latent phase (LIC), single HHV-6A/B or HHV-7 infection in active phase (ASI), double (HHV-6A/B + HHV-7 and HHV-7 + B19V) infection in active phase (ADI), triple (HHV-6A/B + HHV-7 + B19V) infection in an active phase (ATI) and without infection (WI) are depicted in Figs. 6 a–d, respectively. ANOVA test P value shows a statistically significant difference between TNF-α (p = 0.0492), IL-12 (p = 0.0063), and IL-10 (p = 0.0023) levels between the five above-mentioned infection groups, though no difference was detected in the level of IL-6 (p = 0.1289).

According to the cytokine detection protocols, the mean (range) of IL-6 level in apparently healthy individuals was 6.4 (< 0.92–13) pg/ml and IL-10–10.3 (8.1–12.5) pg/ml. TNF-α and IL-12 level in apparently healthy individuals was under the detection level of < 2.3 pg/ml and < 2.1 pg/ml, respectively.

### Severity of ME/CFS in patients with infection/co-infection

**Fig. 7:**
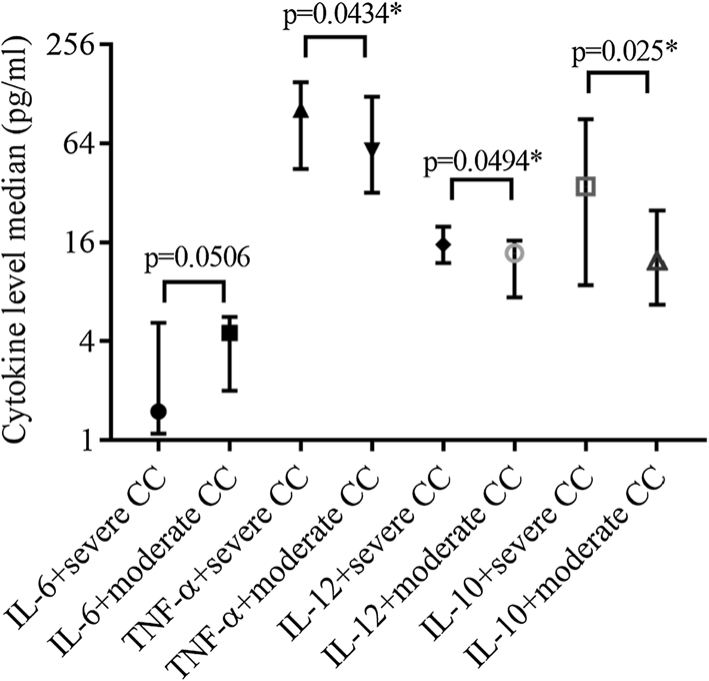
Median (IQR) IL-6, TNF-α, IL-10 and IL-12 level in ME/CFS patients with severe and moderate course of the disease. * statistically significant, *CC* clinical course

A severe course of the disease was experienced by 18.7% and moderate–by 81.3% of patients with ME/CFS (p < 0.0001). In patients with a severe ME/CFS a median (IQR), the IL-6 level was 1.5 (5.2–1.1) pg/ml, TNF-α–103 (150.7–44.7) pg/ml, IL-12–19.9 (37.3–15.5) pg/ml and IL-10 level was 35 (90–8.8) pg/ml. However, in patients with moderate severity of ME/CFS median (IQR), IL-6 level was 4.5 (5.7–2) pg/ml, TNF-α–58 (123–32) pg/ml, IL-12–13.8 (16.4 –7.4) pg/ml and IL-10 level was 12.4 (25–6.7) pg/ml (Fig. 7).

**Fig. 8:**
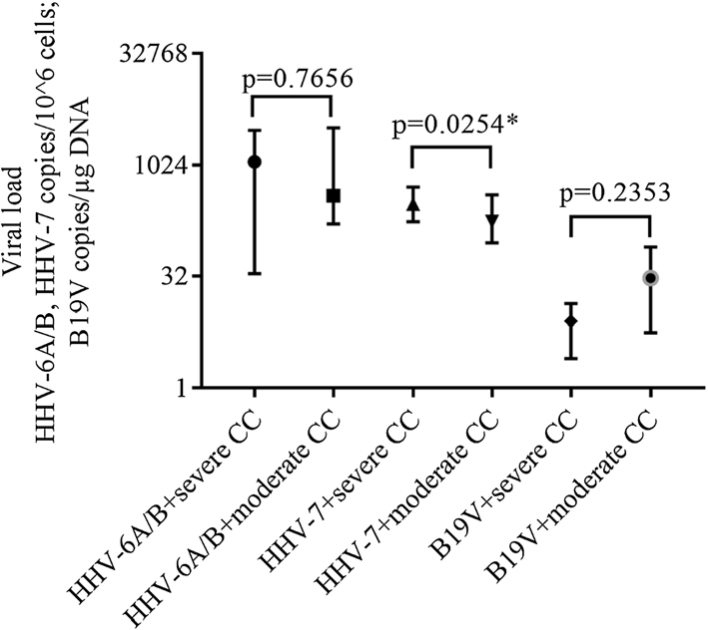
Median (IQR) HHV-6A/B, HHV-7 (copies/10^6^ cells) and B19V (copies/µg DNA) load in ME/CFS patients with severe and moderate course of the disease. * statistically significant, *CC* clinical course

Median (IQR) HHV-6A/B load in ME/CFS patients with severe course of the disease was 1134 (2962–34.5) copies/10^6^ cells and with a moderate course–391.8 (3190–162.8) copies/10^6^ (p = 0.7656). HHV-7 load in patients with severe ME/CFS was 303.6 (514.8–174) copies/10^6^ cells and with a moderate disease course–175.7 (402.7–90) copies/10^6^ cells (p = 0.0254). B19V load in cases of severe and moderate ME/CFS was 8 (13.5–2.5) copies/µg DNA (53 copies/10^6^ cells) and 30 (79.1–5.6) copies/µg DNA (197.8 copies/10^6^ cells), respectively (p = 0.2353) (Fig. 8).

## Discussion

Viral infections are believed to be potential triggers for ME/CFS since infectious-like symptoms are present in many of the ME/CFS patients during the sudden onset of the disease. Severe fatigue can be the consequence of a post-viral infection and immunological dysfunctions may be caused or facilitated by viral infection in patients with ME/CFS. However, there is no consensus on the implication of viral infection in ME/CFS [10].

ME/CFS is more prevalent in females compared with males. Likewise, in this study (65%), it was reported that 65% up to 80% of adult females have ME/CFS [11]. The onset of ME/CFS can occur at any age, though is characteristic to the age of 10 to 19 years and 30 to 39 years [31]. This corresponds to an average age of adult patients in our study (38 ± 12 years).

The frequency of B19V-specific IgG class antibodies is equal in ME/CFS patients and healthy individuals (p = 0.6803), whereas IgM class antibodies are present only in patients with ME/CFS (p = 0.0038). Other B19V seroprevalence studies likewise showed a similarity between the presence of B19V-IgG class antibodies in patients and controls, repoting a B19V seroprevalence of 60–80% [17, 32].

Our study reveals that HHV-6A/B seropositivity is 92.1% of the patients with ME/CFS and 76.7% of the healthy individuals indicating a difference between the groups. Whereas IgM class antibodies are detected in 6.1% of the patients and in only 2.2% of the controls (p = 0.2227). Results on the of HHV-6A/B antibodies prevalence published by other researchers are discrepant. Some report a higher frequency of IgM class antibodies among patients with ME/CFS (50%) compared with healthy donors (28.5%), while others do not find any difference between the patients and the control groups [33–35]. Despite potential differences in geographic distribution, the prevalence of HHV-6A/B IgG class antibodies in healthy adults (76.7%) from this study corresponds to those previously published in Greece (78.8%) [36]. However, Ablashi et al. detected IgM class antibodies more frequently in ME/CFS patients (57.1%) than in healthy donors (16%) in comparison with our study (6.1% and 2.2%, respectively) [15].

In this study, HHV-7 specific IgG class antibodies were detected in 84.6% of ME/CFS patients and in 93.8% of the healthy individuals corresponding to HHV-7 seroprevalence of around 90% among the worldwide adult population [37]. Some researchers find HHV-7 specific antibodies in 91.4% of patients and 88% of controls, whereas some in all ME/CFS patients and 88% of controls [15, 38].

Serological findings could distinguish subgroups of ME/CFS according to the trigger of the disease, however, necessity of multiple testing could be an obstacle [39].

Analysing HHV-6A/B /HHV-7/B19V in this study, persistent infection/co-infection is more frequently found in patients with ME/CFS (96.5%) than the healthy individuals (85.3%) (p = 0.0003). Persistent infection/co-infection in latent phase is revealed in half of the patients with ME/CFS (51.1%) and three quarters of the healthy individuals (76.7%) (p < 0.0001). However, persistent infection/co-infection in active phase is present significantly more often in patients (45%) than in healthy individuals (8.7%) (p < 0.0001), showing the relevance of an active viral infection in ME/CFS. Though, the role of a persistent infection in the latent phase cannot be excluded from studies searching for the trigger factors of ME/CFS and factors influencing disease pathogenesis.

HHV-6A is detected in only one patient with ME/CFS showing that HHV-6B is prevalent among ME/CFS patients in Latvia. Similarly, in another study, HHV-6B is more present in patients with ME/CFS (75%) than HHV-6A (9.7%) [34]. Sairenji et al. find both HHV-6A and HHV-6B antibody titers higher in patients than in controls [38]. In other studies HHV-6A is more prevalent in patients with ME/CFS but HHV-6B ‒ in controls [15, 40]. The differences in distribution of HHV-6A an FFV-6B can be explained by the geographic location, because another study in Latvia also reports on the detection of HHV-6B in Latvian patients with other diseases, like autoimmune thyroiditis [41].

Notably, a single B19V infection is detected in only one patient and in two healthy individuals from this cohort. Considering that HHVs can be helper viruses for replication of the subfamily of parvoviruses – dependoviruses, hypothetically HHV could serve as trigger for B19V infection [42].

The data of this study demonstrate that persistent double HHV-7 + B19V infection in active phase is observed in significantly more patients compared with healthy individuals (p = 0.0002). Moreover, active double HHV-6A/B + HHV-7 and active triple co-infection is found only in patients with ME/CFS (p < 0.0001 and p < 0.0001, respectively), distinctly indicating the involvement of the active co-infection in the development of ME/CFS. Patients with a persistent infection/co-infection in the active phase have a significantly frequently elevated HHV-6A/B and HHV-7 load compared to the latent phase (p = 0.0028 and p = 0.0209, respectively). HHV-6A/B and B19V load is significantly higher in patients with infection/co-infection in the active than in the latent phase (p = 0.0251 and p = 0.0444, respectively). In addition, HHV-7 load is higher in patients with severe compared to the moderate course of ME/CFS, therefore it could be linked with symptoms severity (p = 0.0254). Published analysis of blood DNA viral loads according to co-infections are scarce. Researchers find a similar tendency of higher HHV-7 prevalence and load, as well as higher B19V frequency in patients with ME/CFS compared to controls suggesting B19V involvement in ME/CFS [43]. Changes of HHV-6A/B and HHV-7 salivary DNA load correlate with symptoms of the disease linking pathogenesis to HHV reactivation state [12].

A limited number of studies have analysed the presence of several co-infections in ME/CFS, moreover, most of them do not distinguish the latent from active infections [13, 38], while others find a single EBV infection in an active phase [44]. However, even incomplete HHV-6A/B reactivation can induce secretion of activity producing mitochondrial fragmentation and antiviral response [45]. Many authors have analysed the presence of HHV co-infection and only a few of them have included B19V in co-infection analysis [15, 43]. HHV-6A/B /EBV/B19V co-infection is reported in around 17% of both patients and controls, whereas HHV-7 is observed in a majority of analysed individuals [43]. In this study, we have observed a similar tendency where all co-infections are accompanied by HHV-7, therefore the findings of this study support the hypothesis of ME/CFS as a result of an infectious disease for at least a subgroup of the patients [11].

It is hypothesized that ME/CFS could be caused by neurotropic viruses, like HHV-6A/B and HHV-7, which can infect neurons and immune cells to impair CNS capillaries and micro-arteries, leading to a brain damage. The infection initiates the immune system disturbances that can in turn lead to a chronic infection. Immunosuppression and activated immune complexes may cause chronic inflammation, which facilitates the establishment of persistent infection. Furthermore, chronic immune system activation is accompanied by alterations in the regulation of cytokine production. The abundance of cytokines in plasma significantly correlates with T cell metabolism and is unique in ME/CFS cases [46–48].

The inconsistency among studies on cytokine levels in patients with ME/CFS is explained by variations in patients and controls recruitment in terms of diagnostic criteria, onset, duration, and phase of the disease, as well as the time of sample collection and the laboratory methods used [20]. Increased level of several cytokines after exertion are reported in patients with severe symptom flares [49]. In addition, a disease duration of more than three years is reported to impact the immune signatures [50]. It is shown that HHV-6A/B can infect monocytes/macrophages and inflammatory cytokines can contribute to the reactivation of this virus from a latent phase [18]. Viral infection induced prolonged state of immune disbalance accompanied by changes in cytokine level then can lead to the development of ME/CFS clinical symptoms.

HHV-6A/B immunosuppressive effect on CD4 + lymphocytes involve the suppression of IL-12 expression in dendritic cells. The infectious organism can promote abnormal immune response, though after its elimination immune system changes maintain and cause symptoms of ME/CFS. Interferon-gamma and IL-10 are declared to be sufficient markers for HHV-6A/B induced cell response [51]. The report has also been published on the association of active HHV-6A/B and HHV-7 infection with elevated levels of IL-12 and TNF-α [52]. Moreover, B19V non-structural protein also stimulates such pro-inflammatory cytokines as IL-6 and TNF-α production causing local inflammation [53].

No difference is confirmed in the level of IL-6 among patient groups without infection, with latent infection/co-infection, active single, double and triple co-infection (p = 0.1289). Though a significant difference is revealed in the levels of TNF-α, IL-12, and IL-10 among the five above-mentioned groups (p = 0.0492, p = 0.0063, and p = 0.0023, respectively). Our study results are in accordance with those in the literature on equally raised IL-6 levels without any difference between patients’ and donors’ group [54]. Other researchers also report no differences or even reduced level of IL-6 between ME/CFS and control cases [50, 55, 56]. Further analysis discloses a higher level of IL-6 in patients with active double co-infection than in patients with latent infection/co-infection and without infection (p = 0.0319 and p = 0.0418, respectively). Despite the fact that the level of IL-6 is elevated only slightly, the results show differences among patients with persistent co-infection in the latent and in the active phase, which can be observed only by analysing certain ME/CFS patients’ groups with co-infection. Other studies also report a raised level of IL-6 in patients with ME/CFS [57]. Considering that the average onset of ME/CFS among patients included in this study is 10.2 ± 4.2 months, discrepant results can be explained by a difference in the duration of the disease. Findings of high IL-6 level concern older patients with a duration of ME/CFS for more than two years but a low level of IL-6 concerns younger patients with a recent occurrence of disease (early disease) [19].

Particularly higher level of TNF-α is found in patients with active triple co-infection if compared to latent infection/co-infection and active double co-infection, presenting a role of active co-infection with multiple viruses in an increase of TNF-α level, which indicates an inflammation that could be caused by a viral infection (p = 0.0045 and p = 0.0158, respectively). A study by Brenu and co-authors shows a higher level of TNF-α in ME/CFS patients, however, some authors do not find any difference in TNF-α concentration between patients and controls [54, 55, 57, 58]. Others show reduced TNF-α levels and no association between TNF-α and fatigue severity, however higher levels are accociated with cognitive and musculoskeletal disorders in patients with ME/CFS [56, 59]. Level of TNF-α is lower in long-duration ME/CFS, compared to recent onset of the illness [50]. It is shown that low-level inflammation and activation of cell-mediated immunity are observed in ME/CFS cases and the high level of TNF-α correlates with several clinical symptoms, therefore an increase of inflammatory mediators might explain why these disease symptoms exist [60].

Similarly, in the case of active triple co-infection, the level of IL-12 is more elevated than in latent infection/co-infection, active single, and active double infection cases (p = 0.0003, p = 0.0125, p = 0.0195, respectively). The same tendency in IL-12 level is observed between patients with active triple co-infection and without infection (p = 0.0636). An elevated level of IL-12 is admitted to have a good biomarker potential in ME/CFS [57]. Russell and co-workers also record an increased expression of IL-12 in their study [19]. On the contrary, elsewhere a decreased level of IL-12 is reported in patients compared to controls [61, 62]. Level of IL-12 tends to reduce in long-lasting ME/CFS [50].

Significantly higher level of IL-10 is observed in patients with active double co-infection compared to patients without infection and with latent infection/co-infection (p = 0.029 and p = 0.0035, respectively). In addition, ME/CFS patients with active triple co-infection have a higher level of IL-10 in comparison to patients without infection, with latent infection/co-infection and active single infection (p = 0.0107, p = 0.0034 and p = 0.0321, respectively). The same tendency of IL-10 level increase in patients with ME/CFS is presented in several studies [54, 56, 61]. However, some researchers find a similar level of IL-10 in patients and controls, but some find a statistically significant reduction in level of IL-10 in ME/CFS cases compared tohealthy controls [50, 55, 63, 64]. IL-10 has an immunosuppressive effect and it is associated with chronic infection. In ME/CFS patients IL-10 correlates negatively with a cluster of differentiation – CD8 + T cell glycolysis [48].

Immune dysregulation in ME/CFS patients shows evidence of an autoimmune disease [65]. Thus, cytokines can serve as markers for virus-induced changes in cell immunity and an elevated level of certain cytokines can be associated with inflammation caused by a virus infection [51]. Broderick et al., demonstrate existing immune disturbances in ME/CFS caused by complex networks of cytokine co-expression [46].

Seventeen various cytokines correlate with ME/CFS severity, of which most are pro-inflammatory [66]. It is proved, that the level of pro-inflammatory cytokines correlates with the severity of ME/CFS and sleep disturbances [67]. Besides, the level of TNF-α in patients with ME/CFS correlates with a degree of fatigue [6]. In our study levels of TNF-α, IL-12 and IL-10 are statistically significantly higher in patients with a severe course of ME/CFS compared to those with a moderate course (p = 0.0434, p = 0.0494 and p = 0.025). Inversely, the level of IL-6 tends to be higher in patients with moderate severity of the disease (p = 0.0506). Stress and fatigue are estimated to be greater in patients with an elevated level of IL-6 [68]. A moderate course of ME/CFS is experienced by most of the patients in this study (81.3%) which could be because of the level of IL-6, which is not significantly elevated.

It is possible that a virus infection causes a cellular immunity dysfunction, which induces virus reactivation. Subsequently, viral proteins facilitate cytokine secretion, resulting in the emergence of ME/CFS typical symptoms, such as fatigue, fever, sleep, and cognitive disorders [6, 66]. Chronic pain can be caused by inflammatory signals that are spread by glial cells, whereas inflammatory cytokines and neuronal stimulation can activate glial cells [47]. Recently ME/CFS is described as autoimmune autonomic nervous system imbalance [69].

Besides chronic fatigue for more than 6 months, which all patients with diagnosed ME/CFS have, impaired memory, decreased concentration, and sleep disturbances are the most frequently observed symptoms in these patients. The presence of typical ME/CFS symptoms is reported more frequently among patients in the Netherlands and the United Kingdom. Besides these symptoms, cognitive dysfunction, sleep disturbances, and post-exertional malaise are most frequently reported and are acknowledged to be essential symptoms of ME/CFS [70].

Considering ME/CFS heterogeneity, the use of biomarkers will enable to define subtypes of the disease. Longitudinal and standardized studies determining phenotype and measures of ME/CFS course and therapy effectiveness with follow-up measurements in dynamics should be accomplished. This will allow the prognosis of the disease development and promote the development of a specific definition for diagnostics and a treatment plan (7).

## Conclusions

1. HHV-6A/B, HHV-7, and B19V persistent co-infection in the active phase is significantly more widespread among patients with ME/CFS compared to healthy donors and is characterized by a higher viral load and level of cytokines in comparison to the latent phase of infection. Therefore, markers of HHV-6A/B, HHV-7, and B19V infection could be used as one of the biomarkers in ME/CFS diagnostics.
2. Persistent HHV-6A/B, HHV-7, and B19V co-infection in the active phase might significantly influence the elevation of pro-inflammatory and anti-inflammatory cytokine levels, which can lead to immune disturbances and the development of ME/CFS symptoms.
3. A higher HHV-6A/B and HHV-7 load and a significantly elevated level of pro-inflammatory cytokines TNF-α, IL-12, and anti-inflammatory cytokine IL-10 in patients with a more severe ME/CFS clinical course advocate the involvement of these viral infections in ME/CFS development.

## Data Availability

The datasets used and/or analysed during the current study are available from the corresponding author on reasonable request.

## Abbreviations

B19V: Human parvovirus B19
ELISA: Enzyme linked immunosorbent assay
EBV: Epstein-Barr virus
HHV: Human herpesvirus
Ig: Immunoglobulin
IL: Interleukin
IQR: Interquartile range
ME/CFS: Myalgic encephalomyelitis/chronic fatigue syndrome
nPCR: Nested polymerase chain reaction
PCR: Polymerase chain reactions
SD: Standard deviation
